# Improving an Interprofessional Shared Mental Model and Mutual Understanding Within the Early Links of the Chain of Survival

**DOI:** 10.7759/cureus.104671

**Published:** 2026-03-04

**Authors:** Chelsea Grant, Cristina Iturrrey, Kayla Batista, Mark Yassa, Abigail Alorda, Shayne Gue, Ayanna Walker

**Affiliations:** 1 Emergency Medicine, University of Central Florida (UCF)/HCA Florida Healthcare Graduate Medical Education (GME) (Greater Orlando/Osceola), Kissimmee, USA; 2 Medical Education, University of Central Florida (UCF)/HCA Florida Healthcare Graduate Medical Education (GME) (Greater Orlando/Osceola), Kissimmee, USA; 3 Medical Education, University of Central Florida (UCF) College of Medicine, Orlando, USA; 4 Emergency Medicine, BayCare Health System/St. Joseph's Hospital, Tampa, USA; 5 Emergency Medicine, University of Central Florida (UCF)/HCA Florida Healthcare Graduate Medical Education (GME) (Greater Orlando/Osceola), Orlando, USA

**Keywords:** cardiac arrest, emergency medical services (ems), interprofessional communication, out-of-hospital cardiac arrest, shared mental model

## Abstract

The concept of the "chain of survival" emphasizes a series of critical steps that collectively improve outcomes for patients experiencing cardiac arrest. In practice, these links - dispatchers, emergency medical services (EMS), and emergency department (ED) physicians - often function independently but require integration and collaboration to provide the highest care for our patients. The current study aims to establish an interprofessional shared mental model to improve out-of-hospital cardiac arrest outcomes.

Basic life support (BLS) needs assessments were administered to dispatchers, fire-based EMS crews, and emergency medicine (EM) resident physicians. After the assessments were administered, a focus group was scheduled and performed with dispatch, EMS crews, and EM resident physicians. Once all focus groups were completed and transcribed, a thematic analysis was performed by hand by four researchers to identify the main themes of each focus group. Themes were then compared between the three groups to find commonalities and differences. After establishing common themes, an educational activity was developed and implemented to establish a shared mental model of the chain of survival. The educational activity included EMS crews, EM resident physicians, and EM attending physicians. An interprofessional debrief was then conducted with EM residents, attending physicians, and EMS crews.

A total of 78 individuals completed the needs assessment and participated in focus groups (dispatchers: n = 5; EMS: n = 60; EM residents: n = 13). Thematic analysis of transcribed focus group sessions revealed unique and overlapping challenges across groups, including communication barriers, emotional impact, and environmental limitations. Shared strengths included a mutual understanding of the importance of high-quality cardiopulmonary resuscitation (CPR) and mechanical CPR device utility. Post-curriculum survey responses from 53 EMS participants indicated strong support for the intervention: 71.7% "strongly agreed" that the activity was beneficial, and 75.5% believed it would positively affect daily operations.

Creating a shared mental model through role-specific assessment, interprofessional education, and simulation can bridge gaps in the chain of survival. Improved mutual understanding among dispatchers, EMS personnel, and ED physicians may enhance team coordination and ultimately improve outcomes in cardiac arrest management.

## Introduction

The American Heart Association identifies the concept of "out-of-hospital chain of survival" as a series of critical steps that collectively improve outcomes for patients experiencing an out-of-hospital cardiac arrest. These components include early recognition and activation of emergency response (dispatchers), early cardiopulmonary resuscitation (CPR) (bystanders and emergency medical services (EMS)), rapid defibrillation (EMS), effective advanced life support (EMS and hospital teams), and integrated post-cardiac arrest care (hospital teams) [1]. However, in practice, these components often function independently, with limited integration or collaboration across the chain. This lack of cohesion can hinder the overall effectiveness of the system. Our study aim worked to identify the areas where cohesion was lacking and to create a shared mental model where each chain link participant had an improved shared mutual understanding of the roles and responsibilities of each participant.

Mathieu et al. describe the notion of how "shared mental models help explain how teams are able to cope with difficult and changing task conditions" and mention that shared mental models are crucial to team functioning, especially in situations where communication is difficult [2]. A shared mental model encouraging each individual chain link to understand each other's goals and roles within a resuscitation can allow providers to have a more cohesive approach in an out-of-hospital cardiac arrest. A mixed-methods qualitative analysis reviewing team communication patterns in emergency resuscitation situations identified that team members involved in a resuscitation have a mutual understanding of the goals and roles of resuscitation in regard to providing rapid, life-saving care as well as a need for situational awareness [3]. This analysis review confirmed that many collaborators are involved with information sharing during a resuscitation and addressed how interprofessional communication occurs during these situations during resuscitation in an emergency department [3]. Team coordination, understanding of each other's roles, and communication between team members are the foundation of a shared mental model for the early links of the out-of-hospital chain of survival. Applying these ideas of a shared mental model to the pre-hospital setting and involving dispatchers and EMS providers is where our study was established.

While limited prior data exist regarding the development of focus groups, prior studies regarding the use of shared mental models in clinical scenarios have shown encouraging results. An in-hospital study by Beck et al. focused on mean scores of a team assessment scale as well as hands-off time during a simulated cardiac arrest scenario after being randomized into a conventional versus interventional basic life support (BLS) training group. The interventional group that aimed to develop shared mental models had significantly less hands-off time when compared to the conventional group. This study supports that a shared mental model can not only be effective for team trainings but may also lead to improved team performance, which may result in improved patient outcomes [4]. Similarly, a prospective randomized controlled trial performed by Friederich et al. demonstrated that the development of a shared mental model positively impacted the acquisition of non-technical skills in learners participating in BLS courses as well as demonstrated the effectiveness of simulation-based medical education in the development of technical skills in clinical learning environments [5].

A study completed in a Norwegian university hospital emergency department transcribed audio and video recordings of 10 critical patient situations and evaluated the interconnections between team talk and actions [5]. This study identified that "sharing mutual understanding is crucial for patient safety," although further studies are needed to directly relate communication to patient outcomes [6]. Based on these studies pertaining to in-hospital cardiac arrests and the evaluation of teamwork, communication, and a shared mental model for an interprofessional approach to a patient, we aimed our study to work toward establishing a shared mental model and improving shared mutual understanding by providing needs assessments and engaging in focus group discussions with dispatchers, EMS crews, and emergency medicine (EM) residents.

## Materials and methods

Our interprofessional shared mental model for improving cardiac arrest outcomes was conducted within the St. Cloud Fire Rescue Emergency Medical System, St. Cloud Dispatch Center, and HCA Florida Osceola Emergency Medicine Residency. St. Cloud, Florida, is a suburban community located in Central Florida in Osceola County with a land area of 17.76 square miles. St. Cloud has a total population of 58,964 as of the census in April 2020. St. Cloud Fire Rescue is the local fire rescue agency that services most of St. Cloud, with an annual call volume of about 7,500. St. Cloud Fire Rescue is a fire-based EMS system containing four fire stations in total, each station housing approximately one engine and one rescue. Of the total call volume, there were 119 cardiac arrest calls in 2024.

Prior to focus group sessions, role-specific BLS and needs assessments were administered to dispatchers, EMS personnel, and EM resident physicians. These assessments included both shared and profession-specific questions developed by the authors (Appendices A-F). Questions used in the needs assessments for each group were created and written by the authors and focused on BLS interventions and recommendations as published by the American Heart Association, the interactions between each link in the chain of survival, and the awareness these groups have of one another's roles, needs, responsibilities, and obstacles faced. A five-point Likert scale was used in the needs assessments to evaluate mutual understanding between groups. After the assessment was administered, a focus group was scheduled and performed with dispatch, EMS, and EM resident physicians. Focus groups were conducted with two to three of the authors as facilitators, lasted approximately 60 minutes each, and were structured with open-ended prompts regarding understanding of other groups' roles and responsibilities based on needs assessment responses. Our study was determined to be exempt or excluded from Institutional Review Board (IRB) oversight in accordance with current regulations and institutional policy. All participants provided verbal informed consent prior to recorded focus group participation.

The breakdown of our participants’ numbers by title can be seen in Table 1. Our dispatch group contained a total of 29 full-time dispatchers. Of these 29 dispatchers, 18 needs assessments were completed and five participants were involved in the focus group session. The EMS group consisted of 37 paramedics and 23 emergency medical technicians (EMTs) for the needs assessment and focus group. Our EM resident group consisted of a total of 21 residents. Of these 21 residents, 13 completed the needs assessment and participated in the focus group session.

**Table 1:** Breakdown of participants by job title, total number of eligible participants for each title, number that completed the needs assessment, and number that participated in a focus group session. EMTs: emergency medical technicians; EM: emergency medicine

	Eligible	Completed needs assessment	Completed focus group
Dispatchers	29	18	5
EMTs	31	23	23
Paramedics	58	37	37
EM residents	21	13	13

Once all focus groups were completed and transcribed, a thematic analysis via inductive analysis was performed by hand by four of the authors to identify the main themes of each focus group. Themes were then compared between the three groups to find commonalities and unique differences.

We then utilized these themes from the needs assessment to develop an educational activity for EMS personnel, integrating a shared mental model for the chain of survival. This educational activity involved a simulated out-of-hospital cardiac arrest with transport and hand-off to the receiving hospital where EM residents were working on shift. After this educational activity was completed, an interprofessional debrief occurred with members from EMS as well as hospital staff (EM residents and attendings). A total of 53 paramedics and EMTs completed this educational activity and participated in the interprofessional debrief. Of the 53 participants, 31 of the participants were paramedics and 22 were EMTs.

## Results

The needs assessment questions relating to understanding the roles and responsibilities of dispatch, EMS, and the emergency department were reviewed by the authors. Each independent group primarily answered "agree" or "strongly agree" in regard to describing the role and responsibility of their own group. However, it was noted that there was a higher number of answers of "disagree" and "strongly disagree" on the statements related to the role and responsibility of other domains.

In completing the thematic analysis of the three focus group transcriptions, there were commonalities between the three groups in both positive factors and challenges. There were also noted to be unique positive factors and challenges for each group. A common theme for challenges between the three groups was related to emotion, whether it be from the caller, on-scene family, or family present in the emergency department. A common theme in regard to positive factors was the mutual understanding of the importance and benefit of high-quality CPR in cardiac arrest patients.

Dispatcher group

Our group of dispatchers had a majority of themes related to communication and emotional response when participating in a cardiac arrest situation (Table 2).

**Table 2:** Identified themes in the dispatch focus group with supporting quotes.

Theme	Specific quote - dispatch
Emotional stress impacting caller behavior and response	“Caller is concerned about hurting the patient”; “Sometimes too, callers are in denial about the patient’s status”
Difficulty with identifying cardiac arrest versus other medical emergencies	"There are a lot of time people who are calling for an overdose, but they will not be forthcoming with that information, so we do process a lot of cardiac arrests that end up being overdoses”; “The caller does not always tell us everything that is happening on scene”
Protocols with scripts and perceived benefit of reducing human error	"Even if you are nervous or you have someone who is upset, that because it is scripted, it will forge you the opportunity that as long as you are able to read it, you are not going to lose your place"

Challenges

One of the greatest challenges for our dispatch group was processing and handling emotion from the caller while still trying to provide tele-CPR instructions and dispatch units to the patient as quickly and efficiently as possible. Another challenge that was brought up during our focus group discussion that was unique to dispatch was the difficulty in identifying a true cardiac arrest over the phone in patients who may be experiencing another medical emergency such as an overdose. Our dispatch group pointed out that the caller does not always disclose that the patient has used a substance resulting in an overdose. This challenge affects dispatch in the aspect that they do not always have all of the information available to relay to EMS crews.

Positive Factors

A largely identified positive factor that was unique for our dispatch group was related to having protocols with a script for the dispatcher to follow. It was perceived that this could decrease human error by prompting the dispatcher with the questions to ask based on the caller’s answers. Another positive factor identified was the understanding of the importance and benefit of high-quality CPR. Many of our dispatchers involved in the focus group discussion communicated that they understand that these situations are very time-sensitive and that every second counts.

EMS group

Our EMS group had themes related to emotion as well as location of the patient and themes related to perceived benefits of mechanical CPR devices (Table 3).

**Table 3:** Identified themes in the EMS focus group with supporting quotes. EMS: emergency medical services; CPR: cardiopulmonary resuscitation

Theme	Specific quote - EMS
Emotional impact and communication with family on scene	"I’ve had to talk to family members about us remaining on scene because they’re like, 'Why aren’t you at the hospital? Why are you still here?'"; "It’s always difficult talking to the family while you’re in the middle of a code. We try to be as clear and honest as possible without making them feel hopeless"
Mechanical CPR device perceived benefits and advantages	"The Lucas helps minimize pauses quite a bit. In the past, you would lose a lot of time by transferring the patient and there would be times where you couldn’t perform compressions"; "The Lucas device allowed for more consistent CPR with less interruptions. All of my saves have been from high-quality CPR"
Understanding the benefit and importance of high-quality CPR	"Our crews generally we all know each other very well, so I would be very comfortable saying that if one of us or someone else is having a problem, we have no problem speaking up"; "High-quality CPR consists of minimizing interruptions, compression depth, and rate"

Challenges

During our focus group discussion with the EMS group, one of the most common challenges brought up was related to emotion from the family as well as the physical location of the patient and the surrounding environment. Many EMS personnel commented on the challenges faced when family members on scene are extremely emotional and how it can distract the crews from performing their duties. Another frequently identified theme was related to the location of the patient and the surrounding environment. EMS crews reported that sometimes the location of the patient is a small area where they may not be able to operate as well when compared to being in a more open area or location.

Positive Factors

A positive factor that was frequently mentioned in the EMS group was related to the understanding of the importance and benefit of high-quality CPR. Many of the EMS crew members reported comfort with BLS skills, and many of the senior crew members reported they felt extremely comfortable with instructing and correcting less-senior crew members on techniques related to high-quality CPR, such as the compression rate and compression depth. Another positive theme was the perceived benefit of mechanical CPR devices. Many of the crews reported that mechanical CPR devices are helpful for them, especially when moving a patient to a different location, for example, from the house into the ambulance for transport. There were reports that many times, the transition of patient location would result in a period of time where compressions were not being performed due to the infeasibility of performing compressions while moving the patient. There was a perceived benefit in allowing for significantly decreased pauses in compressions due to having the mechanical CPR device in place.

EM resident group

Our EM group expressed themes related to emotion, communication, and the importance of high-quality CPR (Table 4).

**Table 4:** Identified themes in the EM focus group with supporting quotes. CPR: cardiopulmonary resuscitation; EM: emergency medicine; EMS: emergency medical services

Theme	Specific quote - EM
Communication challenges in cardiac arrest scenarios	"Everybody is talking, no one is doing anything, bystander effect comes into play"; "Sometimes gathering information in the moment as you are trying to take care of the patient, collecting things like down time, any history of the patient, it is difficult to get from EMS in the moment where you are also setting up to take over care of that patient"
Negative impact of CPR interruptions on patient care	"If you think about how many times we stop compressions to put in central line, to intubate, to do pulse checks, to change the Lucas device. All that time adds up and it does increase the mortality"; "Decreasing the compression fraction is bad. We have to stay on the chest at all times as much as possible. Minimizing those interruptions is very important"
Perceived benefits of a mechanical CPR device	“Using the Lucas device that is well-placed is great. It helps minimize the amount of manpower you need because you do not have to have people in line for compressions. A well-placed Lucas is very helpful in cardiac arrest”

Challenges

One of the frequently mentioned themes in our EM focus group was related to crowd dynamics during a cardiac arrest. It was noted that many times when the patient arrived at the ER, nursing staff were beginning to obtain additional access sites and switch the patient over to the ER monitor, all while the lead physician was taking report from the lead paramedic. It was perceived that this prevented the full team from hearing the report and would result in multiple people talking during the resuscitation scenario, which could lead to missed information. Another challenge faced by the EM group was related to the emotion experienced during a cardiac arrest. It was noted that a cardiac arrest is an emotional experience, especially if family members are present in the room during resuscitation efforts. 

Positive Factors

A positive theme during the EM group discussion was related to the importance and benefit of high-quality CPR. Many members voiced the understanding of maintaining a high chest compression fraction and the importance of minimizing interruptions in compressions. An additional positive factor that was identified by the EM group was the perceived positive benefit of mechanical CPR devices.

After our focus group sessions were completed, we conducted a post-educational activity that involved the EMS group and a part of the EM resident group. A short post-activity survey was conducted after the simulation was completed. A total of 53 paramedics and EMTs completed this educational activity and participated in the interprofessional debrief. The overall survey results were positive. In the question of “this activity benefited me,” a total of 38 responses were “strongly agree” (71.7% of total answers). In the question of “I believe this activity will have a positive effect on our daily operations,” a total of 40 participants answered “strongly agree” (75.5% of total answers). We also compared the EMS group needs assessment responses (Table 5) to the post-activity survey responses (Table 6).

**Table 5:** EMS group needs assessment responses EMS: emergency medical services

EMS needs assessment statement	Strongly disagree percentage	Strongly agree percentage
I can describe the role of dispatch in a cardiac arrest	11.70%	31.70%
I can describe the role of the emergency department in a cardiac arrest	3.30%	48.30%

**Table 6:** EMS post-activity survey responses EMS: emergency medical services

EMS post-activity survey statement	Strongly disagree percentage	Strongly agree percentage
I can describe the role of dispatch in a cardiac arrest	0%	64.20%
I can describe the role of the emergency department in a cardiac arrest	0%	66%
I have a better understanding of the different roles within the chain of survival	0%	67.90%

## Discussion

The aim of the current study was to work toward establishing a shared mental model and improved mutual understanding among each component of the chain of survival using needs assessments and focus groups within the community of St. Cloud, Florida. While the results regard reported improvement of mutual understanding of different roles in the chain of survival, these results may not be generalizable to the general population, as the educational curriculum was tailored to the specific needs of the study population. This does represent a limitation to the current study. Another limitation identified in our study was the uneven numbers for focus groups, with dispatch having the smallest group, which could result in biased results. However, similar educational curricula could be developed based on focus groups conducted in other locations to produce similar benefits in those communities. An additional limitation of the current study was not having dispatch involved in the educational simulation activity.

After conducting focus groups among each component of the chain of survival, we came to understand the unique strengths and challenges faced by each group. Among dispatchers, participants cited difficulty making quick decisions regarding the initiation of CPR with callers. Dispatchers also faced an additional challenge in that protocols within the dispatch system must be followed exactly; therefore, individual dispatchers have limited control over what information is relayed to callers. These protocols exist to reduce human error but can contribute to a sense of limitation within individual dispatchers and do not always reflect the dynamics of the situation.

Among EMS personnel, challenges were noted pertaining to the physical location of the patient and the need to move them prior to initiating high-quality CPR. EMS personnel described difficulties “moving a patient from between a bathtub and a toilet” and performing high-quality CPR while “six guys plus the patient” are confined in the limited space of a bathroom. The use of the LUCAS mechanical compression device was cited as a strength among EMS personnel. Our EMS group has a protocol relating to the administration of the LUCAS device after four to five minutes of hands-on compressions. However, an identified challenge this study revealed was switching the EMS group’s LUCAS device to the hospital's device. A process of LUCAS device exchange at the emergency department was developed as a result of this identified challenge.

EM physicians cited several unique challenges pertaining to the environment of the emergency department. Several physicians noted crowd dynamics as a significant factor in the emergency department, as there can often be 10 or more people in the room during a cardiac arrest. This volume of personnel was said to “make it difficult to hear” important information conveyed by others and to “get the story straight” during handoffs with EMS crews. Physicians also noted that department preparedness for a cardiac arrest may vary from day-to-day. Participants stated busier days in the emergency department would often limit the availability of staff and supplies, while slower days would function more smoothly.

Based on the findings, as one component of the curriculum, we developed and implemented an educational simulation activity tailored to optimize cardiac arrest handoffs, which concluded with an interprofessional discussion-style debrief involving both EMS and emergency department teams. A significant limitation of this portion was the lack of dispatch involvement in the simulation activity. Future studies could incorporate dispatch into educational activities to improve mutual understanding. Our initiative aimed to foster collaboration, identify perceived understanding between groups, and lead to improvement of the shared mental model across the chain of survival. Future studies could study the relationship between improved mutual understanding and shared mental models to improve patient outcomes.

While the various groups reported factors unique to their role in the chain of survival, they also shared some common strengths and challenges during the management of patients in cardiac arrest. Common challenges cited among each of the three focus groups were managing the emotion that comes along with a cardiac arrest and difficulty obtaining accurate patient information. The common strength reported among all focus groups was the mutual understanding of the importance of high-quality CPR with minimal interruptions. There is still a significant need for future studies investigating the benefits of a shared mental model and improved mutual understanding within the chain of survival, not only from a teamwork standpoint but also from a patient outcome standpoint.

## Conclusions

Development of a shared mental model and mutual understanding between dispatchers, EMS personnel, and EM physicians regarding cardiac arrest management facilitates patient care. Through the use of needs assessments, focus groups, and a simulation, we were able to work toward establishing a shared mental model between key components of the chain of survival. Our results demonstrate the improved familiarity of dispatchers, EMS personnel, and EM physicians with the various components of the chain of survival after completing an educational curriculum tailored to their specific strengths and challenges. Our hope is that this improved familiarity will have a positive impact on patient care throughout our community.

## Figures and Tables

**Figure 1:**
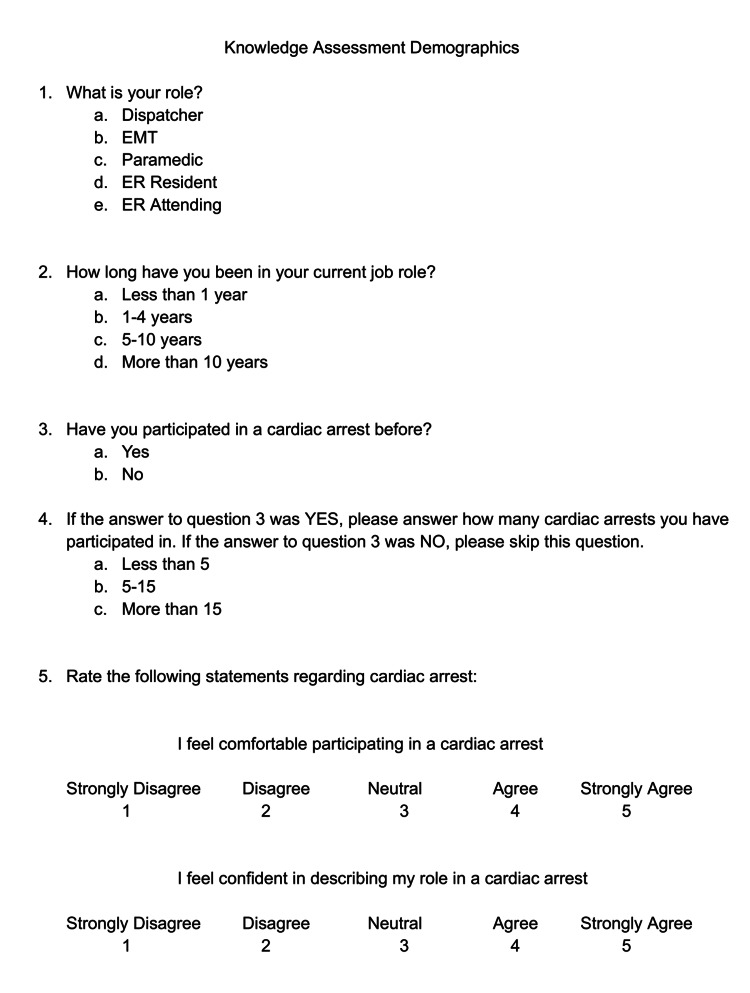
Demographics page 1 administered to all participants. EMT: emergency medical technician

**Figure 2:**
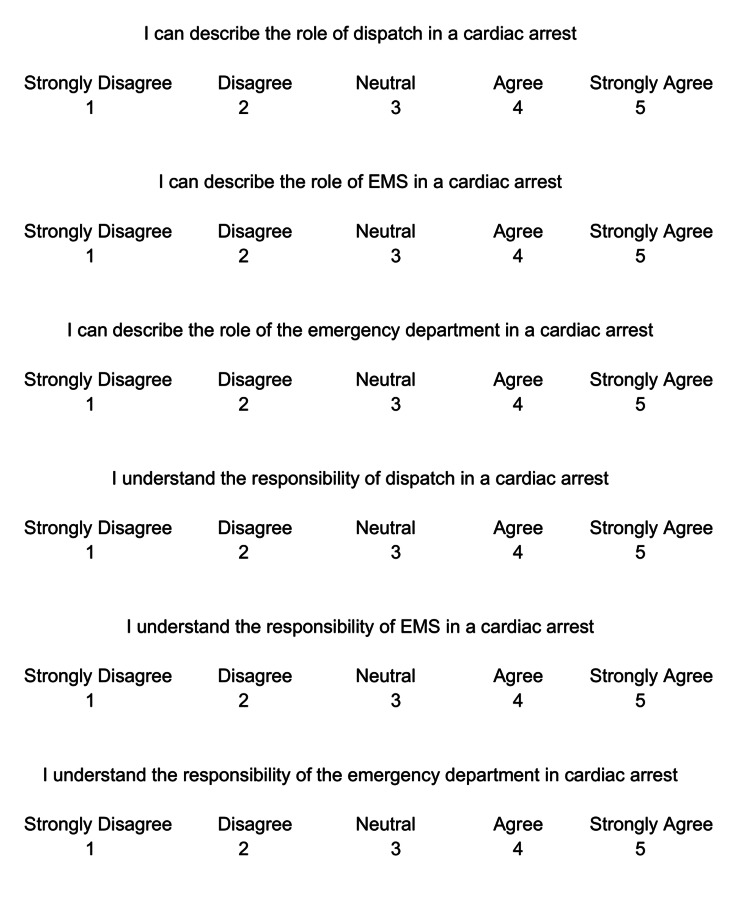
Demographics page 2 administered to all participants. EMS: emergency medical services

**Figure 3:**
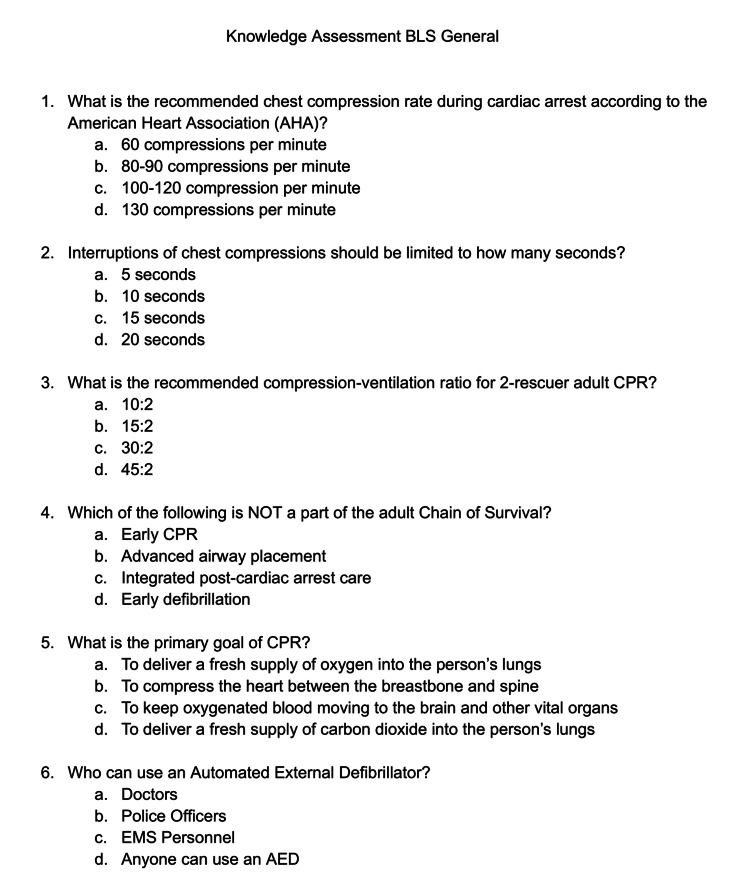
General basic life support (BLS) questions administered to all participants. CPR: cardiopulmonary resuscitation; EMS: emergency medical services; AED: automated external defibrillator

**Figure 4:**
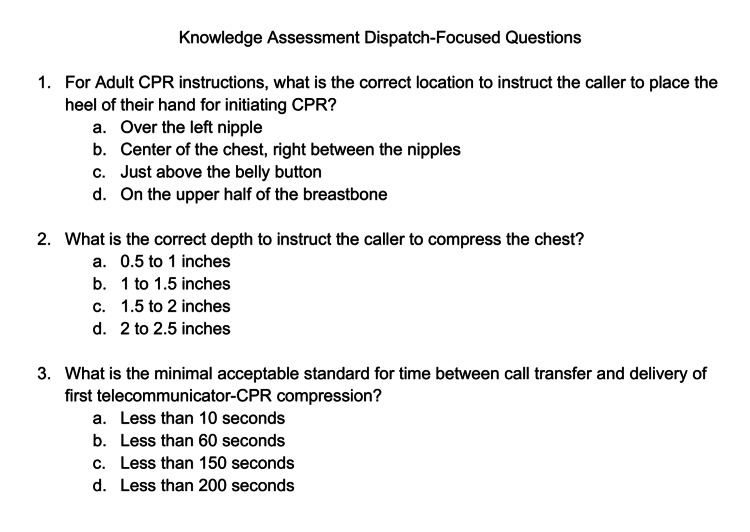
Dispatch-focused questions administered to participants in the dispatch group. CPR: cardiopulmonary resuscitation

**Figure 5:**
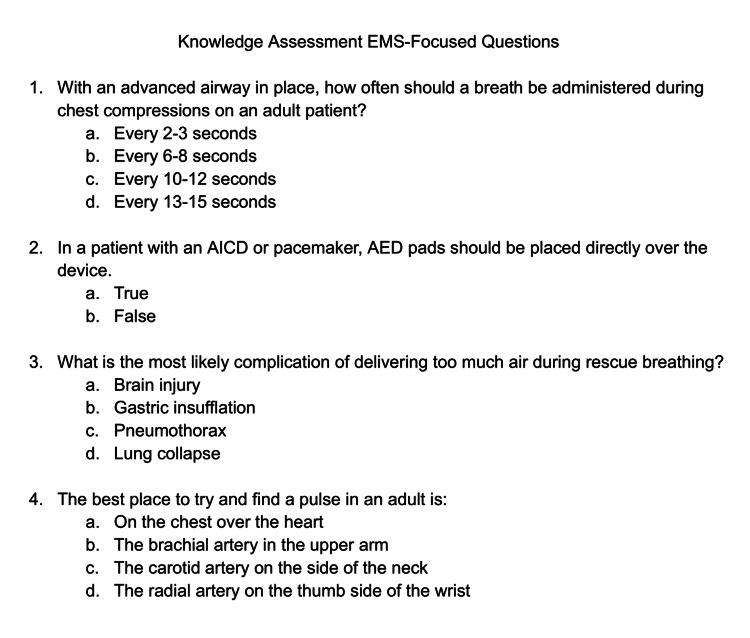
EMS-focused questions administered to participants in the EMS group. EMS: emergency medical services; AICD: automatic implantable cardioverter defibrillator; AED: automated external defibrillator

**Figure 6:**
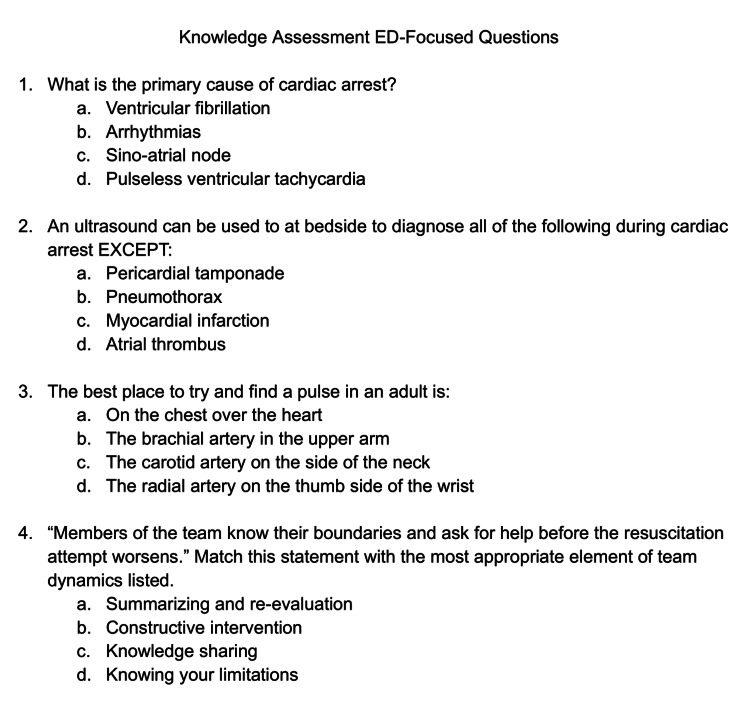
EM-focused questions administered to participants in the EM group. EM: emergency medicine

## Appendices

Appendix A

Appendix B

Appendix C

Appendix D

Appendix E

Appendix F
